# Medication burden in the first 5 years following diagnosis of type 2 diabetes: findings from the *ADDITION-UK* trial cohort

**DOI:** 10.1136/bmjdrc-2014-000075

**Published:** 2015-10-01

**Authors:** James A Black, Rebecca K Simmons, Clare E Boothby, Melanie J Davies, David Webb, Kamlesh Khunti, Gráinne H Long, Simon J Griffin

**Affiliations:** 1 MRC Epidemiology Unit, University of Cambridge, Cambridge, UK; 2 Department of Cardiovascular Sciences, Leicester Royal Infirmary, Leicester, UK

**Keywords:** Medication, Screening

## Abstract

**Introduction:**

Individuals with screen-detected diabetes are likely to receive intensified pharmacotherapy to improve glycaemic control and general cardiometabolic health. Individuals are often asymptomatic, and little is known about the degree to which polypharmacy is present both before, and after diagnosis. We aimed to describe and characterize the pharmacotherapy burden of individuals with screen-detected diabetes at diagnosis, 1 and 5 years post-diagnosis.

**Methods:**

The prescription histories of 1026 individuals with screen-detected diabetes enrolled in the *ADDITION-UK* trial of the promotion of intensive treatment were coded into general medication types at diagnosis, 1 and 5 years post-diagnosis. The association between change in the count of several medication types and age, baseline 10-year UK Prospective Diabetes Study (UKPDS) cardiovascular disease (CVD risk), sex, intensive treatment group and number of medications was explored.

**Results:**

Just under half of individuals were on drugs unrelated to cardioprotection before diagnosis (42%), and this increased along with a rise in the number of prescribed diabetes-related and cardioprotective drugs. The medication profile over the first 5 years suggests multimorbidity and polypharmacy is present in individuals with screen-detected diabetes. Higher modeled CVD risk at baseline was associated with a greater increase in cardioprotective and diabetes-related medication, but not an increase in other medications.

**Conclusion:**

As recommended in national guidelines, our results suggest that treatment of diabetes was influenced by the underlying risk of CVD. While many individuals did not start glucose lowering and cardioprotective therapies in the first 5 years after diagnosis, more information is required to understand whether this represents unmet need, or patient-centered care.

**Trial registration number:**

CNT00237549.

**Key messages**

- Screen-detected diabetes is usually asymptomatic, but individuals often have multimorbidity.
- Just under half of individuals with screen-detected diabetes are on drugs not related to cardiovascular disease at diagnosis.
- Many individuals did not start glucose lowering medication in the 5 years after diagnosis.

## Introduction

Medication burden is high among individuals with established type 2 diabetes. Results from a systematic review indicate that patients with diabetes take in the range of 4 to 10 medications a day.^1^ In an American study of 875 individuals with diabetes, 50% reported taking seven or more prescription medications a day.^2^ Estimates from English patients with diabetes suggest an average of six medications a day.^3^ Individuals with diabetes are prescribed a number of cardioprotective drugs, but there is also evidence to suggest high levels of prescription of other drug classes for example, treatment for neuropathy,^4^ depression,^5^ and gastric and rheumatological symptoms.^6^ In 2012–2013 in England, 9.3% of the total cost of prescriptions in the National Health Service (NHS) was related to diabetes.^7^ As treatment regimens become more complex, patients are more likely to experience adverse side effects^8^ and less likely to remain adherent to all prescribed medications.^9^ ^10^

Less is known about treatment burden among individuals with screen-detected or recently diagnosed diabetes. Given that population screening for diabetes has been recommended by several national organizations and the NHS currently includes assessment of risk of diabetes in its Health Checks program,^11^ more individuals will be found earlier in the disease trajectory. Further, there is growing evidence for the benefit of intensive treatment of risk factors early in the course of the disease,^12^ ^13^ which suggests that screen-detected patients may be prescribed a larger number of cardioprotective drugs earlier than they might previously have been. Although there is some evidence that improved medication adherence may improve health-related quality of life in symptomatic patients with diabetes,^14^ ^15^ individuals earlier in the disease trajectory are unlikely to have symptoms and may be less likely to adhere to complex medication regimes.^16^ ^17^ There is currently little knowledge of medication burden in people with screen-detected diabetes, many of whom will have few or no symptoms. Guidelines promote a multifactorial approach to diabetes care from diagnosis that includes pharmacotherapy for multiple cardiovascular disease (CVD)-related conditions.^18^ ^19^ Despite the increasing number of individuals with screen-detected diabetes, many of whom have comorbidities, there is an absence of knowledge about what the pharmacotherapy burden is at diagnosis in this population, and how it changes in the first 5 years. It is important that this is described so that patients and practitioners are informed about the likely course and burden of treatment. We aimed to (1) describe medication burden at diagnosis, 1 and 5 years in individuals with screen-detected diabetes and (2) examine if age, sex, intensive treatment or modeled 10-year CVD risk was associated with the number of drugs individuals were prescribed at 5 years after diagnosis.

## Methods

The *ADDITION* study is a primary care-based screening and intervention study for type 2 diabetes (ClinicalTrials.gov, CNT00237549). It was carried out in Denmark, the Netherlands and two UK centers (Leicester and Cambridge). The study has been described in detail elsewhere.^13^ ^20^ ^21^ In this paper we describe data from the two UK centers. Briefly, individuals aged 40–69 years in Leicester were invited to attend an Oral Glucose Tolerance Test (OGTT). Individuals in Cambridge aged 40–69 years with a high risk of diabetes in Cambridge (Cambridge Risk Score^22^ ≥0.17) were invited to a stepwise screening program including a random capillary glucose test and glycated hemoglobin, followed by a fasting capillary glucose test and a confirmatory OGTT. Individuals were diagnosed using the WHO 1999 definition of diabetes.^23^ Exclusion criteria included pregnancy, lactation, an illness with a likely prognosis of less than 1 year or a psychiatric illness likely to limit study involvement or invalidate informed consent. Individuals found to have diabetes were treated according to the group to which their practice was allocated: routine care using national guidelines^19^ or the promotion of intensive multifactorial treatment. In the intensive treatment group, general practitioners (GPs) were encouraged through guidelines, educational meetings and audits with feedback to introduce a stepwise target-led drug treatment regime to reduce hyperglycemia, hypertension and hyperlipidaemia^20^ ^21^ similar to the STENO-2 study.^24^ Trained staff-assessed patients’ health at baseline, 1 and 5 years and collected biochemical and anthropometric data according to standard operating procedures. Self-report questionnaires were used to collect information on sociodemographic information, lifestyle habits and medication use. The study was approved by the relevant ethics committees^13^ ^20^ ^21^ and all participants provided written informed consent.

### Assessment of medication

In Cambridge, participants were encouraged to bring their repeat prescription summaries to each health assessment and self-reported medication was collected via a health economics questionnaire which asks for information on all prescribed medication.^25^ In Leicester, prescription information could also be sourced directly from the records of a peripatetic clinic. Medication data were coded using the Anatomical Therapeutic Chemical Classification System (ATC).^26^ ATC codes were used to derive counts for each participant within the following 23 classes of medication: insulin, metformin, sulphonylurea, thiazolidinediones, other glucose lowering medication, ace-inhibitors, β-blockers, calcium channel blockers, diuretics, other blood pressure lowering medications, lipid lowering, antithrombotic, gastrointestinal-related, skin-related, hormone-replacement therapy or urogenital, systemic steroids, thyroid-related, anti-inflammatory, analgesic, antiepileptic, psychiatric, respiratory and eye-related. Medication counts in this analysis refer to the number of the 23 classes prescribed (not overall pill count), while medication agent refers to 1 of the 23 explored classes of medication. For several analyses, these 23 categories were also collapsed into diabetes-related (insulin, metformin, sulphonylurea, thiazolidinediones, other glucose-lowering medication), cardioprotective (ace-inhibitors, β-blockers, calcium channel blockers, diuretics, other hypertension-related medications, lipid-lowering, antithrombotic) and other (remaining 11 classes). Medication types that were not within these categories, for example acute medications like antibiotics, were not included in these analyses.

### Statistical analysis

Baseline and 5-year descriptive characteristics of the cohort were summarized using means, medians and proportions. We described the medication profile of the *ADDITION-UK* cohort at diagnosis, 1 and 5 years following diagnosis. Using complete case linear regression, we explored the mutually adjusted associations between age, baseline 10-year UK Prospective Diabetes Study (UKPDS) CVD risk,^27^ sex, treatment group and baseline number of medications on (1) change in total number of medications, (2) change in cardioprotective medications and (3) change in other medications between diagnosis and 5 years. Owing to the distribution of change in diabetes-related medication being left-censored at zero an analogous Poisson regression model was used to explore the association between baseline predictors and change in diabetes-related medication over 5 years. SEs were used to adjust for clustering by GP practice in the models. As current guidelines for the treatment of type 2 diabetes are very similar to the protocol used in the intensive treatment arm of *ADDITION-UK,* and the achieved difference in treatment was small, treatment arms were pooled for the primary analysis.^13^ ^28^ A sensitivity analyze by randomization arm showed little differences relative to overall changes.

In order to characterize missing data, we used logistic regression models to derive the odds of being included in the complete-case analysis, individually adjusted for age, sex, baseline UKPDS 10-year CVD risk, treatment group and 2004 indices of multiple deprivation (IMD). IMD scores were only available for the 867 individuals (86% of the sample) from the Cambridge area, so the association between missing data and socioeconomic status is described using a smaller data set for this sensitivity analysis.

The small differences in the outcome and treatment between routine care and intensive treatment in *ADDITION-Europe* has been linked to the continual improvement of routine care, most likely accelerated through the introduction of the Diabetes National Service Framework in 2001,^29^ clinical guidelines for targeting blood pressure and lipids in people with diabetes in 2002,^19^ and the Quality and Outcomes Framework in 2004.^13^ ^29^ Current guidelines for the treatment of type 2 diabetes are similar to the protocol used in the intensive treatment arm of *ADDITION-UK*.^13^ ^28^ As such, while a statistically significant difference in cardioprotective and glucose-lowering drugs is present, absolute differences in the prevalence of medications being reported are small, which is why treatment arms were pooled in this analysis.

Statistical analyses were performed using R 3.0.2 (checkpoint 2014-09-18).

## Results

At diagnosis, the *ADDITION-UK* cohort had a mean age of 61 years (SD 7), a median UKPDS 10-year CVD risk of 19% (IQR 13, 27) and 61% were male (tables 1 and 2). Of the 1026 individuals in the *ADDITION-UK* cohort, 1024 (99.8%) had medication data at diagnosis, 1008 (99% of living) at 1 year, and 930 (96% of living) at 5 years. Ten people died before 1 year follow-up, and 59 before 5-year follow-up.

**Table 1:** Baseline characteristics of the ADDITION-UK cohort, overall and by previous CVD status and CVD risk quartile

	10-year UKPDS CVD risk: Lowest quartile 5,17	10-year UKPDS CVD risk: Highest quartile 3692	No CVD	Previous CVD*	Total
N†	244	244	858	106	1026
Mean age in years (SD)	55.6 (7.5)	64.2 (5.3)	60.3 (7.5)	63.1 (5.3)	60.6 (7.4)
Male %	40%	83%	60%	74%	61%
White %	80%	98%	93%	96%	91%
Median 10-year CVD risk (IQR)	14 (11, 15)	47 (40, 56)	24 (17, 33)	45 (35, 56)	25 (17, 36)
Mean BMI kg/m^2^ (SD)	32.8 (5.8)	33.0 (5.8)	33.3 (5.7)	32.9 (6.1)	30.8 (5.4)
Mean HbA_1C_ %	6.6 (1.1)	8.3 (2.2)	7.4 (1.7)	7.1 (1.6)	7.3 (1.7)
Mean HbA_1C_ mmol/mol	49 (12)	68 (24)	57 (19)	53 (17)	57 (18)
Mean systolic BP mm Hg (SD)	133 (16)	153 (23)	143 (19)	139 (22)	146 (17)
Mean total cholesterol mmol/L (SD)	5.2 (1.0)	5.5 (1.3)	5.5 (1.1)	4.6 (1.0)	5.6 (1.2)
Self-reported CVD* %	1%	30%	0%	100%	11%
Self-reported high-blood pressure %	60%	55%	57%	68%	59%
Self-reported high cholesterol %	27%	31%	23%	68%	28%

*Previous myocardial infarction or stroke.

†Number of participants recruited at diagnosis.

BMI, body mass index; BP, blood pressure; CVD, cardiovascular disease; HbA1c, glycated hemoglobin; UKPDS, UK Prospective Diabetes Study.

**Table 2:** Association between baseline characteristics at diagnosis and change in medication count between diagnosis and 5 years in the ADDITION-UK cohort

	Change in total medication count		Change in diabetes medication		Change in CVD medication		Change in other medication	
	β*	95% CI	IRR*	95% CI	β*	95% CI	β*	95% CI
Number of medications at diagnosis†	−0.49	−0.56 to −0.42	–	–	−0.50	−0.56 to −0.44	−0.30	−0.37 to −0.22
Male gender	−0.25	−0.57 to 0.06	0.86	0.75 to 0.99	−0.11	−0.33 to 0.10	0.12	−0.10 to 0.34
Intensive treatment arm	0.44	0.10 to 0.78	1.14	1.01 to 1.30	0.39	0.09 to 0.69	−0.08	−0.30 to 0.13
Age at diagnosis (years)	−0.03	−0.05 to −0.01	0.96	0.95 to 0.97	−0.02	−0.03 to 0.002	0.02	0.01 to 0.04
Modelling 10-year UKPDS CVD risk (%)	0.04	0.02 to 0.05	1.02	1.01 to 1.03	0.02	0.01 to 0.03	0.00	−0.01 to 0.01

*IRR, Incidence Rate Ratio from a Poisson regression model; β, Regression coefficient from a linear regression model.

†Number of medications of the medication type that is the dependent variable in that columns regression.

CVD, cardiovascular disease; UKPDS, UK Prospective Diabetes Study.

### Total medication burden

**Figure 1:**
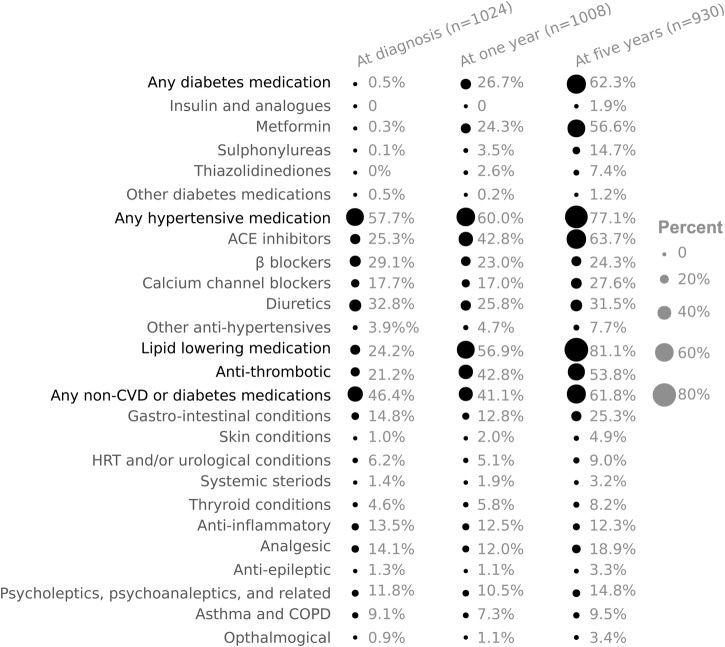
Proportions of self-reported medication use in the ADDITION-UK cohort at diagnosis, 1 and 5 years. COPD, chronic obstructive pulmonary disease; CVD, cardiovascular disease; HRT, hormone replacement therapy.

At diagnosis, most individuals reported taking two medications (median 2; IQR 0, 4). This was most commonly a cardioprotective medication (median 1; IQR 0, 3), although some individuals were on more than one non-cardioprotective medication at diagnosis (figure 1). One year after diagnosis a median of 3 medications (IQR 0,6) were recorded. At 5 years, individuals were typically prescribed six medications (median 6; IQR 5, 8), which included one diabetes-related medication (median 1; IQR 0, 1), four cardioprotective medications (median 4; IQR 3, 5) and one other medication (median 1; IQR 0, 2).

### Diabetes-related and cardioprotective medication

**Figure 2:**
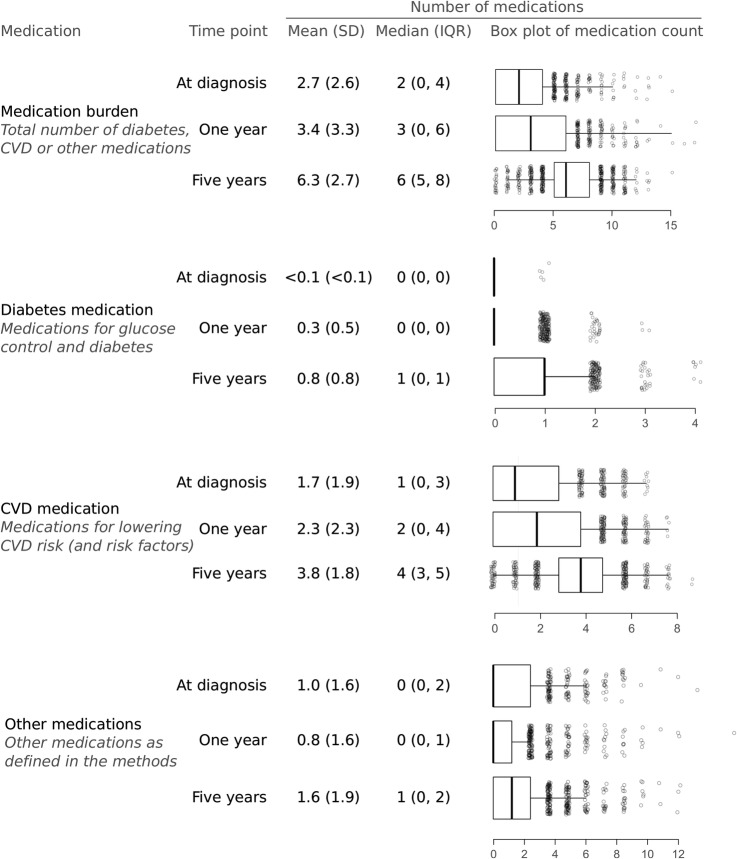
Count of medication types reported in the ADDITION-UK cohort at diagnosis, 1 and 5 years. CVD, cardiovascular disease.

After diagnosis, both the variety and number of cardioprotective and diabetes medications increased (figure 2). At 1 year, 23% of individuals were prescribed any type of diabetes medication, which increased to 62% at 5 years. Between diagnosis, 1 and 5 years, the prescription of antihypertensive (55% to 51% to 77%), lipid-lowering (24% to 48% to 81%) and anti-thrombotic (20% to 36% to 54%) medication increased. In this screen-detected population, many individuals reported using no glucose lowering medication at 1 and 5 years (78% and 38%, respectively, figures 1 and 2).

### Other medications

At diagnosis, 42% of individuals were prescribed other types of medication, which increased to 62% at 5 years after diabetes diagnosis (figure 2). The most common was for gastrointestinal conditions (13% at diagnosis, and 25% at 5 years). Many individuals also reported anti-inflammatory (12% at diagnosis, and 12% at 5 years), analgesic (12% at diagnosis, and 19% at 5 years) and psychotherapy (11% at diagnosis, and 15% at 5 years)-related prescriptions.

### Association between baseline characteristics and number of prescribed drugs at 5 years

The baseline characteristics associated with an increase in the total number of prescribed drugs between diagnosis and 5 years were a younger age (β −0.03, 95% CI −0.05 to −0.01), a higher baseline modeled 10-year UKPDS CVD risk score (β 0.04, 95% CI 0.04, 95% CI 0.02 to 0.05), randomisation to the intensive treatment arm of the trial (β 0.44, 95% CI 0.01 to 0.78), and being prescribed less medications at diagnosis (β −0.49, 95% CI −0.56 to −0.42). Sex was not associated with change in total number of medications. Similarly, the baseline characteristics associated with an increase in cardioprotective medication were a higher 10-year CVD risk (β 0.02, 95% CI 0.01 to 0.02), randomization to the intensive treatment arm (β 0.39, 95% CI 0.09 to 0.69) and being prescribed less medication at baseline (β −0.50, 95% CI −0.56 to −0.44). An increase in diabetes-related medication was associated with female sex (Incidence Rate Ratio, IRR 0.86, 95% CI 0.75 to 0.99), younger age (years; IRR 0.96, 95% CI 0.95 to 0.97), having a higher baseline 10-year CVD risk (IRR 1.02, 95% CI 1.01 to 1.02) and randomization to the intensive treatment arm (IRR 1.15, 95% CI 0.01 to 1.30).

Compared to individuals with medication data at 5 years, those without medication data were more likely to be female (OR 0.56; 95% CI 0.35 to 0.89), older (1 year; OR 0.97; 0.94 to 0.999), to have had a previous CVD event (OR 0.49; 95% CI 0.29 to 0.90) and to be in the intensive arm of the trial (OR 2.04; 95% CI 1.32 to 3.20). There was no association between loss to follow-up and ethnicity (White vs other; OR 0.77; 95% CI 0.31 to 1.60) or socioeconomic deprivation (1 point IMD 2004 change; OR 0.99; 95% CI 0.97 to 1.02).

## Discussion

In a population of individuals with screen-detected type 2 diabetes, we described the prevalence of diabetes-related, cardioprotective and other medications at diagnosis, 1 and 5 years post-diagnosis. Many individuals were on medications not related to cardioprotection before diagnosis (42%), and this increased along with a rise in the number of diabetes-related and cardioprotective drugs. At 5 years, individuals were typically prescribed six medications, including one diabetes-related medication, four cardioprotective medications, and one other medication. This suggests that there is a significant degree of multimorbidity and polypharmacy present in individuals with screen-detected diabetes. Following diagnosis, individuals were more likely to be prescribed diabetes-related medication if they were younger, female, had a high modeled CVD and if they were randomized to the intensive treatment arm of the trial. Younger individuals being prescribed more total and diabetes medication in the 5 years after diagnosis is in line with previous literature that identified those with early diabetes as having worse glycaemic control elevated and CVD risk factors.^30^ In older individuals, the balance between treatment benefits and harm may also become less clear, which could also lead to the identified association. Higher modeled CVD risk at baseline was associated with a greater increase in cardioprotective medication, but not an increase in other medications. As recommended in national guidelines, our results suggest that the treatment of diabetes was influenced by the underlying risk of CVD.

This is the first description of total medication burden in a large cohort of individuals with screen-detected diabetes over 5 years of follow-up. In a subset of the Dutch Hoorn Study, among 195 individuals with screen-detected diabetes, 45% were taking blood-pressure lowering medication and 20% were taking lipid-lowering medication at diagnosis.^31^ In *ADDITION-UK* at diagnosis, 55% of individuals were taking blood pressure-lowering medication, and 24% lipid-lowering medication, in agreement with the results of the Hoorn screening subsample. In a separate publication from the Hoorn study, 2 weeks after diagnosis 24% of the screen-detected and 78% of the clinically detected individuals were prescribed oral glucose-lowering medication.^32^ The step-wise screening program carried out in *ADDITION-Cambridge* used the Cambridge Risk Score to identify those at the highest risk of undiagnosed diabetes.^22^ This score includes blood pressure medication as a variable, which may have led to an overestimate in the number of individuals taking antihypertensive medication in this sample. In 2005–2006, in an American population with long-standing diabetes, 90% of the population were taking glucose-lowering medications, 78% were taking antihypertensives and 26% were on statins.^33^ This contrasts with *ADDITION-UK*, where glucose-lowering medications were less common (62%, at 5 years), and statins were more common (54%, at 5 years). Statin use was the pharmacotherapy that differed by the greatest margin between arms of the *ADDITION-UK* trial (47% for routine care vs 60% after the promotion of intensive care, at 5 years). Our results suggest that the promotion of statin use is the most readily accepted treatment after diagnosis compared to the introduction of glucose-lowering treatment. In ADDITION-Europe, we have previously demonstrated that individuals with the worst cardiometabolic health at diagnosis were the most likely to be prescribed glucose, blood pressure and lipid lowering medication, and also were likely to achieve the greatest reductions in individual CVD risk factors over the 5 years immediately after diagnosis.^34^

Previous literature has noted that the prescription of cardioprotective medication often lags behind glucose-lowering medication, suggesting a disproportionate emphasis on controlling glucose over CVD risk reduction.^33^ ^35^ In both arms of *ADDITION-UK, use of* antihypertensive and lipid-lowering medication was reported by around four-fifths of the participants (77% and 81%, respectively), and glucose-lowering and aspirin use was reported for three-fifths of the population (62% and 54%, respectively). Our results suggest that the prescription of cardioprotective medication did not lag behind that of glucose-lowering. Conversely, 20% of individuals were on metformin at 1 year, and 57% at 5 years, despite metformin being recommended as a first line glucose-lowering medication, and immediate initiation being recommended by National Institute for Health and Care Excellence if overweight or non-responsive to lifestyle interventions.^19^ Variation in treatment could be a positive indicator of patient-centered care or a deficit between patient need and prescribed medication. More detailed knowledge on the circumstances around treatment choices in screen-detected populations would help inform whether the prescription of cardioprotective and glucose-lowering medication should be higher in this population, or that the proportions prescribed medications in this study represent adequate care in relation to GP and patient needs and priorities. An increase in diabetes medication from diagnosis to 5 years was associated with being female, younger, having a GP who was in the trial arm promoted to treat intensively and having a higher baseline risk of a CVD event. In the Hoorn study, 2 weeks after screen-detected diabetes diagnosis, 24% of the population were taking glucose-lowering medication.^32^ While previous literature suggests there is no association between the prescription of diabetes-related medication and gender.^36^ ^37^

### Strengths and limitations

*ADDITION-UK* is a large cohort (n=1026) with consistency in outcome measurement and little loss to follow-up in individuals prescription histories (4% at 5 years). ADDITION-UK (91% white ethnicity) was less diverse than the UKPDS (81% white ethnicity),^38^ which may limit generalisability. However, ADDITION-UK remains the only study able to characterize medication changes after screen-detected diabetes diagnosis while receiving contemporary diabetes care. This analysis uses prescribed medications, which is likely to be an over count of the redeemed and consumed prevalence. Some medications may also be available without a prescription. Accuracy of medication data was improved by encouraging participants to bring repeat prescriptions to the health assessment, the use of a health economics questionnaire^25^ and cross-referencing GP records to collect medication data. For the secondary analysis of change in medications, our analysis assumes that a change from zero to one medication is directly comparable to a change from four to five, or two to one. Medication was coded into 23 classes, but anti-infectives, antiparasitics and antineoplastic medications (as defined by the ATC) were not included as they were acute (eg, infections) or rare (eg, cancer). As this study collected snapshots of medication use at baseline, 1 and 5 years after diagnosis, we are not able to give accurate prevalences for acutely prescribed medications. The number of medical agents was chosen over the raw pill count as some medications can be taken as ‘combination’ pills, or can be split across multiple doses. This could unduly increase the impact of some medications that are taken multiple times a day on the final medication count. There is also likely to be less agreement between the doctor prescribed treatments and daily pill count, compared to reported types of medical agent, as pill count includes both agent and information on frequency and method of dose. Information on non-CVD-related comorbidities that may influence medication was not collected. This analysis remains primarily descriptive, and does not directly assess the relationship between changes in cardiometabolic health and pharmacotherapy. This analysis is unable to describe the pharmacotherapy of individuals that died during follow-up, and it is likely that if medication at the time of death was available, it would introduce greater heterogeneity to this analysis. There was no association between loss to follow-up and change in medication, although this analysis was limited to the subsample of Cambridge participants (86% of the sample) due to the IMD scores not being available for all centers.

Individuals with screen-detected diabetes are often taking multiple medications before diagnosis, despite being identified early in the diabetes disease trajectory. This includes both cardioprotective medications, and other medications including; gastrointestinal, anti-inflammatories, analgesics and psychiatric/neurological medications. After diagnosis, GPs and patients appear to adopt pharmacological strategies that target both CVD risk reduction and glycemia, providing evidence against concerns of over-prioritizing glycemic targets. The increased prescription of cardioprotective medication was associated with higher baseline CVD risk, indicating an association between need and care. While this result is promising, it remains unclear if the prescription rates of glycemic and cardioprotective medication in this population with elevated cardiovascular risk reflect individualized treatment based on patient led priorities or a deficit in the application of pharmacological intervention.
